# 2-[(3*S*)-5-Oxooxolan-3-yl]isoindoline-1,3-dione

**DOI:** 10.1107/S160053681105135X

**Published:** 2011-12-03

**Authors:** Hui Wang, Changlu Liu, Feihua Luo

**Affiliations:** a Department of Chemistry and Chemical Engineering, Sichuan University of Arts and Science, Sichuan Key Laboratory of Characteristic Plant Development and Research, Sichuan Dazhou 635000, People’s Republic of China

## Abstract

The oxolan-2-one ring in the title compound, C_12_H_9_NO_4_, has an envelope conformation with the atom linking the two five-membered rings being the flap atom.

## Related literature

For the synthesis of the title compound, see: Temperini *et al.* (2010 ▶). For the structure of the closely related compound, 2-(2,5-dioxotetra­hydro­furan-3-yl)isoindoline-1,3-dione, see: Qian (2008 ▶).
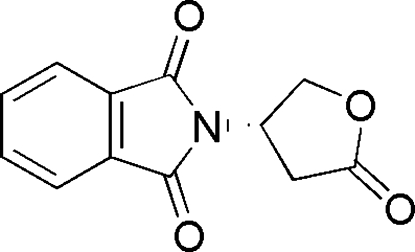


## Experimental

### 

#### Crystal data

C_12_H_9_NO_4_
*M* *_r_* = 231.20
Orthorhombic, 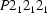

*a* = 5.7224 (3) Å
*b* = 10.5839 (5) Å
*c* = 16.8532 (10) Å
*V* = 1020.72 (9) Å^3^
*Z* = 4
Mo *K*α radiation
μ = 0.12 mm^−1^
*T* = 296 K
0.20 × 0.20 × 0.14 mm

#### Data collection

Bruker SMART CCD area-detector diffractometer
Absorption correction: multi-scan (*SADABS*; Sheldrick, 1996 ▶) *T*_min_ = 0.977, *T*_max_ = 0.984
4468 measured reflections
1077 independent reflections
1002 reflections with *I* > 2σ(*I*)
*R*_int_ = 0.021

#### Refinement

*R*[*F* ^2^ > 2σ(*F* ^2^)] = 0.028
*wR*(*F* ^2^) = 0.073
*S* = 1.20
1077 reflections
154 parameters
H-atom parameters constrained
Δρ_max_ = 0.10 e Å^−3^
Δρ_min_ = −0.13 e Å^−3^

### 

Data collection: *SMART* (Bruker, 2001 ▶); cell refinement: *SAINT* (Bruker, 2001 ▶); data reduction: *SAINT*; program(s) used to solve structure: *SHELXS97* (Sheldrick, 2008 ▶); program(s) used to refine structure: *SHELXL97* (Sheldrick, 2008 ▶); molecular graphics: *PLATON* (Spek, 2009 ▶); software used to prepare material for publication: *SHELXTL* (Sheldrick, 2008 ▶).

## Supplementary Material

Crystal structure: contains datablock(s) I, global. DOI: 10.1107/S160053681105135X/lh5369sup1.cif

Structure factors: contains datablock(s) I. DOI: 10.1107/S160053681105135X/lh5369Isup2.hkl

Supplementary material file. DOI: 10.1107/S160053681105135X/lh5369Isup3.cml

Additional supplementary materials:  crystallographic information; 3D view; checkCIF report

## Figures and Tables

**Fig. 1.**
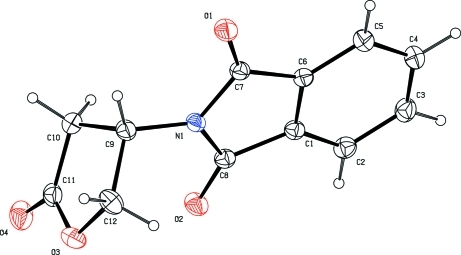
The molecular structure of with displacement ellipsoids drawn at the 20% probability level.

## supplementary crystallographic information

### Comment

The title compound is a key intermediate in our organic synthesis work. It was originally synthesized by Temperini *et al.* (2010). We report herein its crystal structure.

The molecular structure of the title compound (I) is shown in Fig. 1. The structure of the closely related compound, 2-(2,5-Dioxotetrahydrofuran-3-yl)isoindoline-1,3-dione, has already been published (Qian, 2008). In (I) The five-membered ring is in an envelope conformation.

### Experimental

The title compound was prepared from L-Aspartic acid as starting  material. First, the amino  acid was converted into the Cbz-protected lactone. Then, the Cbz-protected lactone (2.34 g, 10 mmoL) was hydrogenated at 1 atm  with phthalic anhydride (1.78 g, 12 mmoL) in 30 mL of DMF and 0.53 g of 10% Pd/C. Single crystals suitable for X-ray diffraction were obtained by evaporation of the a DMF solution of the title compound.

### Refinement

H atoms were placed in calculated positions and treated as riding atoms with C-H= 0.93 - 0.98 Å, with U_iso_(H)=1.2U_eq_(C). In the absense of significant anomalous dispersion effects the Friedel pairs were merged. The absolute  configuration was based on that of the starting material.

### Crystal data

**Table:**

C_12_H_9_NO_4_	*F*(000) = 480
*M**_r_* = 231.20	*D*_x_ = 1.505 Mg m^−^^3^
Orthorhombic, *P*2_1_2_1_2_1_	Mo *K*α radiation, λ = 0.71073 Å
Hall symbol: P 2ac 2ab	Cell parameters from 2599 reflections
*a* = 5.7224 (3) Å	θ = 2.3–27.2°
*b* = 10.5839 (5) Å	µ = 0.12 mm^−^^1^
*c* = 16.8532 (10) Å	*T* = 296 K
*V* = 1020.72 (9) Å^3^	Block, colourless
*Z* = 4	0.20 × 0.20 × 0.14 mm

### Data collection

**Table:**

Bruker SMART CCD area-detector diffractometer	1077 independent reflections
Radiation source: fine-focus sealed tube	1002 reflections with *I* > 2σ(*I*)
graphite	*R*_int_ = 0.021
φ and ω scans	θ_max_ = 25.0°, θ_min_ = 2.3°
Absorption correction: multi-scan (*SADABS*; Sheldrick, 1996)	*h* = −6→6
*T*_min_ = 0.977, *T*_max_ = 0.984	*k* = −12→12
4468 measured reflections	*l* = −19→17

### Refinement

**Table:**

Refinement on *F*^2^	Primary atom site location: structure-invariant direct methods
Least-squares matrix: full	Secondary atom site location: difference Fourier map
*R*[*F*^2^ > 2σ(*F*^2^)] = 0.028	Hydrogen site location: inferred from neighbouring sites
*wR*(*F*^2^) = 0.073	H-atom parameters constrained
*S* = 1.20	*w* = 1/[σ^2^(*F*_o_^2^) + (0.0366*P*)^2^ + 0.0827*P*] where *P* = (*F*_o_^2^ + 2*F*_c_^2^)/3
1077 reflections	(Δ/σ)_max_ < 0.001
154 parameters	Δρ_max_ = 0.10 e Å^−^^3^
0 restraints	Δρ_min_ = −0.13 e Å^−^^3^

### Special details

**Table:**

Geometry. All esds (except the esd in the dihedral angle between two l.s. planes) are estimated using the full covariance matrix. The cell esds are taken into account individually in the estimation of esds in distances, angles and torsion angles; correlations between esds in cell parameters are only used when they are defined by crystal symmetry. An approximate (isotropic) treatment of cell esds is used for estimating esds involving l.s. planes.
Refinement. Refinement of F^2^ against ALL reflections. The weighted R-factor wR and goodness of fit S are based on F^2^, conventional R-factors R are based on F, with F set to zero for negative F^2^. The threshold expression of F^2^ > 2sigma(F^2^) is used only for calculating R-factors(gt) etc. and is not relevant to the choice of reflections for refinement. R-factors based on F^2^ are statistically about twice as large as those based on F, and R- factors based on ALL data will be even larger.

### Fractional atomic coordinates and isotropic or equivalent isotropic displacement parameters (Å^2^)

**Table:**

	*x*	*y*	*z*	*U*_iso_*/*U*_eq_	
C1	0.8388 (3)	0.49330 (17)	0.93941 (11)	0.0362 (4)	
C2	0.6577 (4)	0.5765 (2)	0.95245 (13)	0.0445 (5)	
H2	0.5424	0.5900	0.9145	0.053*	
C3	0.6548 (4)	0.6395 (2)	1.02494 (13)	0.0493 (5)	
H3	0.5347	0.6960	1.0360	0.059*	
C4	0.8280 (4)	0.6193 (2)	1.08081 (13)	0.0501 (5)	
H4	0.8220	0.6627	1.1288	0.060*	
C5	1.0100 (4)	0.5361 (2)	1.06693 (13)	0.0470 (5)	
H5	1.1268	0.5232	1.1044	0.056*	
C6	1.0115 (3)	0.47294 (17)	0.99517 (11)	0.0365 (4)	
C7	1.1748 (3)	0.37695 (18)	0.96411 (11)	0.0366 (4)	
C8	0.8880 (3)	0.41079 (18)	0.87031 (11)	0.0371 (4)	
C9	1.2036 (4)	0.2499 (2)	0.83890 (11)	0.0422 (5)	
H9	1.3392	0.2152	0.8669	0.051*	
C10	1.0378 (4)	0.1429 (2)	0.81692 (13)	0.0500 (6)	
H10A	1.1173	0.0621	0.8192	0.060*	
H10B	0.9051	0.1408	0.8527	0.060*	
C11	0.9611 (4)	0.1714 (2)	0.73468 (13)	0.0449 (5)	
C12	1.2804 (4)	0.2984 (2)	0.75729 (11)	0.0524 (6)	
H12A	1.2911	0.3898	0.7575	0.063*	
H12B	1.4318	0.2639	0.7432	0.063*	
N1	1.0948 (3)	0.34698 (16)	0.88763 (9)	0.0351 (4)	
O1	1.3448 (3)	0.33039 (14)	0.99497 (8)	0.0510 (4)	
O2	0.7776 (3)	0.39782 (15)	0.80953 (9)	0.0510 (4)	
O3	1.1040 (3)	0.25678 (15)	0.70162 (8)	0.0522 (4)	
O4	0.8005 (3)	0.12687 (16)	0.69809 (11)	0.0653 (5)	

### Atomic displacement parameters (Å^2^)

**Table:**

	*U*^11^	*U*^22^	*U*^33^	*U*^12^	*U*^13^	*U*^23^
C1	0.0359 (10)	0.0372 (10)	0.0356 (10)	−0.0026 (9)	0.0013 (9)	0.0020 (8)
C2	0.0405 (11)	0.0454 (11)	0.0476 (12)	0.0047 (9)	0.0003 (10)	0.0038 (10)
C3	0.0514 (12)	0.0423 (11)	0.0543 (13)	0.0063 (11)	0.0120 (11)	−0.0014 (10)
C4	0.0613 (13)	0.0461 (11)	0.0429 (12)	0.0036 (11)	0.0050 (11)	−0.0071 (10)
C5	0.0543 (12)	0.0460 (12)	0.0406 (12)	0.0020 (10)	−0.0040 (10)	−0.0038 (9)
C6	0.0408 (9)	0.0344 (10)	0.0342 (10)	−0.0010 (8)	0.0003 (9)	0.0027 (8)
C7	0.0396 (10)	0.0371 (10)	0.0332 (10)	−0.0022 (9)	−0.0040 (9)	0.0023 (8)
C8	0.0360 (9)	0.0384 (10)	0.0369 (10)	−0.0005 (8)	−0.0034 (9)	0.0024 (8)
C9	0.0442 (10)	0.0453 (11)	0.0371 (11)	0.0112 (10)	−0.0067 (9)	−0.0038 (9)
C10	0.0698 (14)	0.0366 (11)	0.0436 (12)	0.0036 (11)	−0.0023 (11)	−0.0004 (9)
C11	0.0484 (13)	0.0404 (11)	0.0460 (12)	0.0097 (10)	−0.0052 (10)	−0.0094 (10)
C12	0.0452 (12)	0.0686 (16)	0.0435 (12)	−0.0027 (12)	0.0070 (10)	−0.0075 (11)
N1	0.0370 (8)	0.0379 (9)	0.0306 (8)	0.0039 (7)	−0.0028 (7)	−0.0008 (7)
O1	0.0515 (8)	0.0563 (9)	0.0452 (8)	0.0123 (8)	−0.0160 (8)	−0.0030 (7)
O2	0.0479 (8)	0.0621 (10)	0.0430 (8)	0.0088 (8)	−0.0144 (7)	−0.0063 (7)
O3	0.0560 (8)	0.0674 (10)	0.0331 (8)	0.0037 (9)	−0.0010 (7)	0.0007 (8)
O4	0.0620 (9)	0.0601 (10)	0.0737 (11)	0.0063 (9)	−0.0234 (10)	−0.0173 (9)

### Geometric parameters (Å, °)

**Table:**

C1—C2	1.378 (3)	C8—O2	1.211 (2)
C1—C6	1.381 (3)	C8—N1	1.394 (2)
C1—C8	1.482 (3)	C9—N1	1.455 (2)
C2—C3	1.392 (3)	C9—C10	1.523 (3)
C2—H2	0.9300	C9—C12	1.532 (3)
C3—C4	1.383 (3)	C9—H9	0.9800
C3—H3	0.9300	C10—C11	1.484 (3)
C4—C5	1.384 (3)	C10—H10A	0.9700
C4—H4	0.9300	C10—H10B	0.9700
C5—C6	1.382 (3)	C11—O4	1.203 (2)
C5—H5	0.9300	C11—O3	1.340 (3)
C6—C7	1.476 (3)	C12—O3	1.446 (3)
C7—O1	1.208 (2)	C12—H12A	0.9700
C7—N1	1.404 (2)	C12—H12B	0.9700
			
C2—C1—C6	121.98 (18)	N1—C9—C12	113.13 (18)
C2—C1—C8	130.15 (18)	C10—C9—C12	102.07 (17)
C6—C1—C8	107.87 (16)	N1—C9—H9	109.4
C1—C2—C3	117.1 (2)	C10—C9—H9	109.4
C1—C2—H2	121.5	C12—C9—H9	109.4
C3—C2—H2	121.5	C11—C10—C9	105.11 (18)
C4—C3—C2	121.0 (2)	C11—C10—H10A	110.7
C4—C3—H3	119.5	C9—C10—H10A	110.7
C2—C3—H3	119.5	C11—C10—H10B	110.7
C3—C4—C5	121.5 (2)	C9—C10—H10B	110.7
C3—C4—H4	119.3	H10A—C10—H10B	108.8
C5—C4—H4	119.3	O4—C11—O3	121.1 (2)
C6—C5—C4	117.4 (2)	O4—C11—C10	128.7 (2)
C6—C5—H5	121.3	O3—C11—C10	110.14 (18)
C4—C5—H5	121.3	O3—C12—C9	106.29 (18)
C1—C6—C5	121.05 (19)	O3—C12—H12A	110.5
C1—C6—C7	108.61 (16)	C9—C12—H12A	110.5
C5—C6—C7	130.32 (19)	O3—C12—H12B	110.5
O1—C7—N1	124.45 (19)	C9—C12—H12B	110.5
O1—C7—C6	129.62 (19)	H12A—C12—H12B	108.7
N1—C7—C6	105.93 (15)	C8—N1—C7	111.08 (16)
O2—C8—N1	124.41 (18)	C8—N1—C9	125.97 (16)
O2—C8—C1	129.20 (18)	C7—N1—C9	122.54 (16)
N1—C8—C1	106.39 (16)	C11—O3—C12	111.18 (16)
N1—C9—C10	113.28 (16)		
			
C6—C1—C2—C3	−0.3 (3)	C12—C9—C10—C11	−21.9 (2)
C8—C1—C2—C3	178.4 (2)	C9—C10—C11—O4	−165.3 (2)
C1—C2—C3—C4	0.4 (3)	C9—C10—C11—O3	16.2 (2)
C2—C3—C4—C5	0.0 (3)	N1—C9—C12—O3	−101.25 (19)
C3—C4—C5—C6	−0.4 (3)	C10—C9—C12—O3	20.8 (2)
C2—C1—C6—C5	−0.2 (3)	O2—C8—N1—C7	−176.92 (19)
C8—C1—C6—C5	−179.17 (18)	C1—C8—N1—C7	3.3 (2)
C2—C1—C6—C7	178.63 (17)	O2—C8—N1—C9	−4.1 (3)
C8—C1—C6—C7	−0.3 (2)	C1—C8—N1—C9	176.10 (18)
C4—C5—C6—C1	0.6 (3)	O1—C7—N1—C8	176.68 (18)
C4—C5—C6—C7	−177.97 (19)	C6—C7—N1—C8	−3.45 (19)
C1—C6—C7—O1	−177.9 (2)	O1—C7—N1—C9	3.6 (3)
C5—C6—C7—O1	0.8 (4)	C6—C7—N1—C9	−176.58 (16)
C1—C6—C7—N1	2.3 (2)	C10—C9—N1—C8	−52.5 (3)
C5—C6—C7—N1	−179.1 (2)	C12—C9—N1—C8	63.0 (2)
C2—C1—C8—O2	−0.4 (4)	C10—C9—N1—C7	119.54 (18)
C6—C1—C8—O2	178.5 (2)	C12—C9—N1—C7	−124.9 (2)
C2—C1—C8—N1	179.41 (19)	O4—C11—O3—C12	178.82 (19)
C6—C1—C8—N1	−1.7 (2)	C10—C11—O3—C12	−2.6 (2)
N1—C9—C10—C11	100.02 (19)	C9—C12—O3—C11	−12.1 (2)
